# Localization-delocalization transition in spin-orbit-coupled Bose-Einstein condensate

**DOI:** 10.1038/srep31700

**Published:** 2016-08-17

**Authors:** Chunyan Li, Fangwei Ye, Yaroslav V. Kartashov, Vladimir V. Konotop, Xianfeng Chen

**Affiliations:** 1 Key Laboratory for Laser Plasma (Ministry of Education), Collaborative Innovation Center of IFSA (CICIFSA), Department of Physics and Astronomy, Shanghai Jiao Tong University, Shanghai 200240, China; 2 ICFO-Institut de Ciencies Fotoniques, The Barcelona Institute of Science and Technology, 08860 Castelldefels (Barcelona), Spain; 3 Institute of Spectroscopy, Russian Academy of Sciences, Troitsk, Moscow Region, 142190, Russia; 4 Centro de Física Teórica e Computacional and Departamento de Física, Faculdade de Ciências, Universidade de Lisboa, Campo Grande 2, Edifício C8, Lisboa 1749-016, Portugal

## Abstract

We address the impact of the spin-orbit (SO) coupling on the localization-delocalization-transition (LDT) in a spin-orbit coupled Bose-Einstein condensate in a bichromatic potential. We find that SO coupling significantly alters the threshold depth of the one of sublattices above which the lowest eigenstates transform from delocalizated into localized. For some moderate coupling strengths the threshold is strongly reduced, which is explained by the SO coupling-induced band flattening in one of the sub-lattices. We explain why simultaneous Rabi and SO coupling are necessary ingredients for LDT threshold cancellation and show that strong SO coupling drives the system into the state where its evolution becomes similar to the evolution of a one-component system. We also find that defocusing nonlinearity can lead to localization of the states which are delocalized in the linear limit.

Localization of a wave in a one-dimensional linear system obeying translational symmetry, either continuous as in homogeneous space or discrete as in perfectly periodic potentials, is impossible and can be achieved only if the symmetry is broken. In the opposite case of a random systems all the states are localized, what is well known as Anderson localization^1^,^2^,^3^. An intermediate position between translational invariant and random potentials is usually attributed to quasi-periodic lattices, and in particular to bichromatic potentials with non-commensurable periods. Depending on the relation between the periods, such potentials may support localized and delocalized states. The transition between localization and delocalization subject to variation of system parameters is referred to as localization-delocalization transition (LDT).

Predicted more than three decades ago for a tight-binding approximation for incommensurable potentials^4^ and for Aubry-André^5^ (also known as Harper^6^) model^7^,^8^,^9^, LDT recently attracted increasing attention due to its experimental observation in Bose-Einstein condensates^10^ and in optics^11^. In particular, dependence of localization on the degree of commensurability was studied in ref. 12. Extension of the Aubry-André model beyond the tight-binding model bringing it closer to a continuous bi-chromatic potential, has evidenced the appearance of mobility edges reported in ref. 13. The method was suggested that allows to get exact result by extending aperiodic Schrödinger equation to a higher space where a one-dimensional quasiperiodic function with two incommensurate periods is embedded in a two-dimensional space of periodic functions^14^. Furthermore, it was shown that LDT may occur not only in conservative, but also in active incommensurable 1D lattices^15^,^16^, obeying the parity-time symmetry (i.e. where gain and loss are balanced). Particular attention was focused on atomic systems, cold bosonic atoms described by one-dimensional Bose-Hubbard model^17^,^18^. Numerical study of evolution of BECs and dipolar BECs in a bi-chromatic lattices were reported in refs 19,20.

The studies of one-dimensional LDT, mentioned above, dealt with one-component systems. Atomic systems represent a natural platform for studying two– (and multi–)component systems, since they allow one to introduce diverse types of the gauge potentials^21^, which presently are not available in other settings. This in particular is the case of spin-orbit (SO) coupled BECs recently created experimentally^22^ (see also^23^,^24^ for recent reviews). Such condensates are characterized by spinor rather than by scalar wavefunctions with a momentum dependent linear coupling between two components which can be manipulated by external laser fields.

While the effect of the spin-orbit coupling on localization is a problem raised a few decades ago^25^ for electrons in two-dimensional random potentials, there are only a few studies devoted to SO coupled BEC^26^ in incommensurable lattices. In ref. 27 there has been developed a tight-binding model extending the Aubry-André model which includes SO coupling and the existence of the mobility edge was found. The model however did not contain nonlinearity. Further study reported in ref. 28 has considered a BEC in a continuous bi-chromatic model, but with fixed relation between the depth of two lattices, and thus only localized modes were found. Thus to the full extent the impact of SO coupling on LDT has not being explored so far. Meantime the phenomenon reveals several unexpected features, which either cannot be described by the simplified tight-binding model or were overlooked in the analysis of the continuous model. In particular, in this Report we show that SO coupling drastically modifies LDT in continuous bichromatic potentials and under appropriate conditions results in strong reduction of the threshold depth of one of sub-lattices above which localization is observed. We reveal that this effect is connected with band flattening in one of sub-lattices mediated by SO coupling. Meantime very strong SO coupling drives the system into the state where its evolution becomes similar to evolution of one-component system. Finally we found that increase of the defocusing nonlinearity may result in strong localization.

## Results and Discussion

**Model.**  We consider a spinor BEC which in the meanfield approximation is described by the spinor **Ψ** = (*ψ*_1_, *ψ*_2_)^*T*^ (hereafter the upper index *T* indicates the transposed matrix) which is governed by the Gross-Pitaevskii equation (GPE)^22^,^23^,^24^


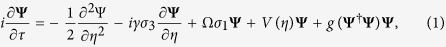


which is written in the dimensionless units where 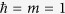, *σ*_1,3_ are the Pauli matrices, *γ* characterizes strength of the spin-orbit coupling, Ω is the dimensionless Rabi frequency, and *g* > 0 is proportional to the inter-atomic scattering length (below we also use *g* = 0 for noninteracting atoms, i.e. for the linear limit). The potential *V*(*η*) represents a bi-chromatic aperiodic optical lattice which we choose in the form


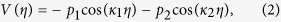


where *p*_1,2_ describe depths of two sub-lattices and *κ*_1,2_ indicate their lattice constants. Without loss of generality we can fix *κ*_1_ = 2. Furthermore, we limit the consideration to 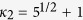, which provides incommensurable lattice periods and is one of the most studied cases in the one-component systems. In particular, it was shown experimentally for single-component BECs and in optics that in the linear case, *g* = 0, such bichromatic lattice *V*(*η*) supports transition from delocalized eigenmodes to localized ones occurring upon variation of only one parameter of the system (for example, depth of the second sub-lattice *p*_2_) for all other parameters being fixed. Bearing this in mind we fix the depth of the first sub-lattice *p*_1_ = 1, unless stated otherwise, leaving *p*_2_ as a control parameter.

We should mention that, in (1) we used the nonlinearity with all inter-atomic interactions being equal. A more general case would imply substitution of 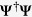 by the diagonal matrix


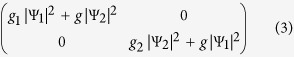


where *g*_1,2_ characterize intra-specie two-body interactions and *g* is the coefficient of inter-specie interaction. It turns out, that typical experimental values of these coefficients may differ within only a few percents. Say in ref. 22 the respective relations are given by: *g*_1_/*g*/*g*_2_ ≈ 0.995/0.995/1 (i.e. *g*_1_ ≈ *g*_2_ ≈ *g*). The results presented below, in particular those depicted in Fig. 5, do not change qualitatively even if this small difference in nonlinearity coefficients *g*_1_, *g*_2_, and *g* is taken into account.

### Mode analysis

In this work we are interested in the impact of spin-orbit coupling on LDT, and respectively we address the stationary solutions having the form 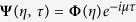 where *μ* is the chemical potential and the stationary spinor **Φ** = (*ϕ*_1_, *ϕ*_2_)^*T*^ solves the stationary GPE, which in the linear limit can be written as:


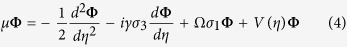


The problem is solved numerically on sufficiently large window 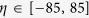 with zero boundary conditions for **Φ**. Since we are interested primarily in specifics of LDT in this system, for characterization of localization degree we introduce the integral form-factor


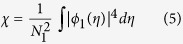


where 
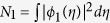
 is the number of atoms in the first component [for dynamically evolving wavepackets the same definition is used with *ϕ*_1_(*η*) replaced by 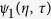], and follow its variation upon modification of parameters *γ* and *p*_2_. The limit *χ* → 0 corresponds to delocalized modes, while form-factor *χ* ~ 1 corresponds to strongly localized states having widths comparable to sub-lattice periods.

The sufficiency of quantity (5) for characterization of the degree of localization of modes of the *two*-component system under consideration stems from its symmetry ensuring that both components have the same degree of localization. Indeed the symmetries of (1), and respectively of (4), imply that if the spinor 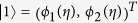 is a solution of (4), then 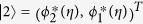, 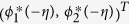, and 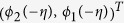 are solutions as well. This readily gives that, if a chemical potential is nondegenerate, i.e. if all these states are equal to each other with a possible constant phase factor, then 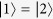 and hence 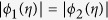. In its turn, this means that form-factors of two components are equal. If any two of the above states are different, i.e. the chemical potential is degenerated, then one can consider a superposition of the states. For example, if 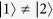 one can consider 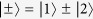 yielding different states but with the same form-factors of the components. Obviously the components of |+〉 have equal modulus distributions, but opposite inhomogeneous phases, while the phases of the components of |−〉 are opposite with the additional constant *π*-phase shift in the second component.

**Figure 1:**
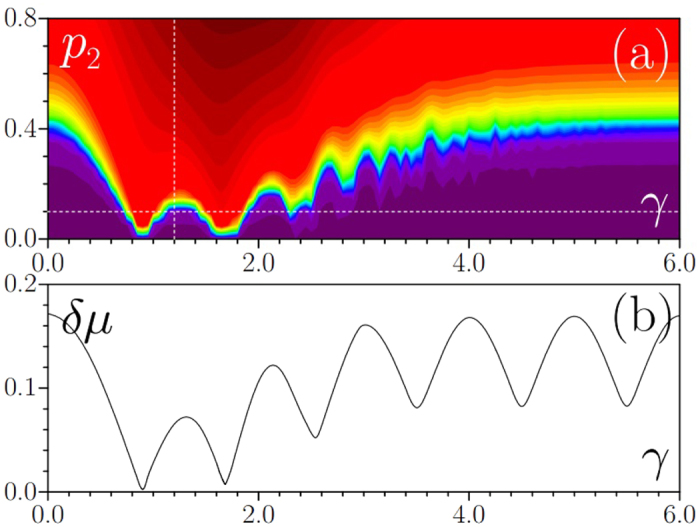
(**a**) Form-factor of eigenmode with lowest chemical potential on the plane (*γ, p*_2_) at *p*_1_ = 1, *κ*_1_ = 2, *κ*_2_ = 5^1/2^ + 1, **Ω** = 1. Dashed lines indicate cross-sections in which eigenmode transformation is shown in Fig. 2. (**b**) Width of the first band in periodic lattice with *p*_1_ = 1, *κ*_1_ = 2 versus spin-orbit coupling strength *γ*.

The linear eigenvalue solver usually returns combination of the above mentioned modes. Among the whole spectrum of (4) we look mainly at solutions with the smallest chemical potentials *μ*, since LDT occurs in fact simultaneously for the simplest mode with lowest *μ* centered at *η* = 0 and for a number of higher-order states with larger *μ* that are typically displaced from the center of potential. Color-scale Fig. 1(a) shows representative dependence of the form-factor of such simplest mode on the depth of the second sub-lattice *p*_2_ and SO coupling strength *γ*. One can see that in all cases LDT occurs when the depth *p*_2_ exceeds a certain threshold value (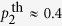 at *γ* = 0). However, our central result is that SO coupling drastically modifies this localization threshold. Unexpectedly, for specific values of *γ*, clearly distinguishable in Fig. 1(a), this threshold nearly vanishes predicting that localization is possible even for a very weak incommensurable modulation of the main sub-lattice. One can observe a rich (and somewhat irregular at large *γ*) structure of deeps in the *χ*(*γ, p*_2_) dependence, indicating that SO coupling sometimes acts towards reduction of localization threshold, but sometimes also towards its increase. Note that at *γ* → ∞ the LDT threshold becomes independent of *p*_2_ and gradually approaches its value at *γ* = 0.

**Figure 2:**
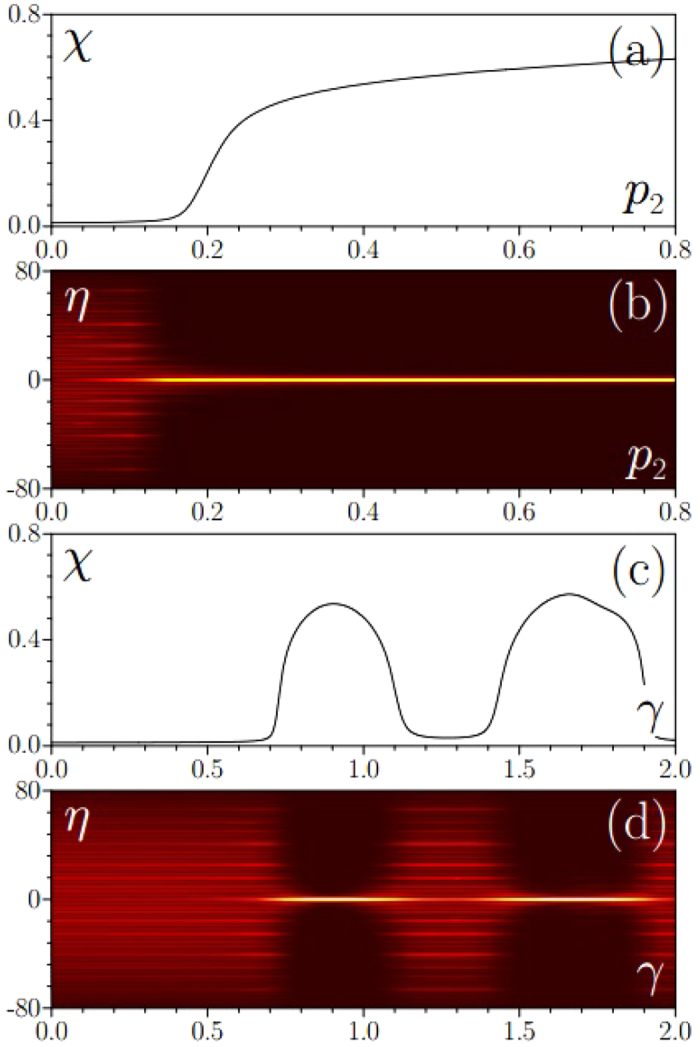
Modification of the form-factor of eigenmode (**a**) and corresponding shape transformation (**b**) with increase of *p*_2_ at *γ* = 1.2. Modification of form-factor of eigenmode (**c**) and corresponding shape transformation (**d**) with increase of *γ* at *p*_2_ = 0.1. Here *p*_1_ = 1, *κ*_1_ = 2, *κ*_2_ = 5^1/2^ + 1, **Ω** = 1.

Transformation of the density distribution |*ϕ*_1_|^2^ of the first spinor component of the linear eigenmode upon variation of *p*_2_ and *γ* is illustrated in Fig. 2 (the density distribution of the second component is identical, as discussed above). One can see from Fig. 2(a,b) that although modes become rigorously localized starting from certain threshold depth of the second sub-lattice, slightly above the LDT threshold they are still strongly extended and transition to patterns occupying just a couple of periods of potential occurs within finite interval of *p*_2_ values. It is thus practical to introduce quantitative criterion for LDT threshold in *p*_2_ or *γ*, by defining it, for example, at *χ* = 0.1 level. One observes that the form-factor of the mode is a monotonically growing function of *p*_2_, but it can show strongly nonmonotonic behavior upon variation of *γ* [Fig. 2(c,d)]. Dependencies shown in Fig. 2 correspond to cross-sections of Fig. 1(a) marked with dashed lines.

In order to understand this behavior it is instructive to consider impact of SO coupling on spatial dispersion introduced by the potential with *p*_2_ = 0 (i.e. its impact on the band-gap spectrum of the perfectly periodic structure involving just one sub-lattice). In this case the system obeys band-gap spectrum and the solution of (4) is a Bloch mode, i.e. 
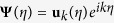
 where 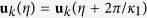 and *k* is the quasimomentum. Figure 1(b) shows dependence of the width of the lowest band in the spectrum of the first sub-lattice defined as 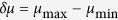, where *μ*_max_ and *μ*_min_ are the upper and lower edges of the band, respectively. We selected the lowest band because the internal structure of the localized mode with smallest *μ* value in the incommensurable potential with 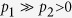 closely resembles that of the Bloch mode from the lowest band of periodic potential (2) with *p*_1_ ≠ 0 and *p*_2_ = 0. One can see from Fig. 1(b) that SO coupling leads to remarkable flattening of the lowest band in the spectrum: bandwidth becomes nearly zero for two specific *γ* values and it oscillates periodically at large *γ*. The effect of band flattening, discussed also in ref. 29, means nearly vanishing spatial dispersion around the points where bandwidth is minimal. Hence even small second sub-lattice should be sufficient to suppress very weak dispersion around these points and may result in the formation of localized states. Indeed, one observes drastically reduced LDT threshold in Fig. 1(a) exactly around first two bandwidth minima in *γ* in Fig. 1(b). For large *γ* the bandwidth does not shrink so strongly and to observe LDT one has to use second sub-lattice with depth *p*_2_ comparable to *p*_1_. Clearly, in this regime spatial dispersion strongly departs from that for single sub-lattice, and multiple “irregular” deeps appear in the *χ*(*γ, p*_2_) dependence in Fig. 1(a).

Further understanding of the LDT can be achieved in terms of the transformed spinor **F**:


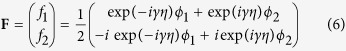


which solves the stationary equation


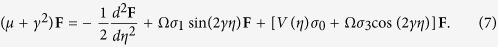


Here *σ*_0_ is a 2 × 2 identity matrix. Since the coefficients of the obtained Eqs. (6) are all real, the components of **F** can be searched real too. In the absence of the linear coupling, i.e. at Ω = 0, the equations for components *f*_1,2_ become decoupled and fully identical even at *γ* ≠ 0. Thus, the behavior of corresponding system becomes identical to that of single-component system without any SO coupling. On the other hand, in the absence of SO coupling, i.e. at *γ* = 0 the system again becomes equivalent to two independent one-component systems in identical bi-harmonic lattices. This explains why for observation of the effects reported here it is crucial to have *both* SO coupling and linear Rabi coupling (this was also a conclusion of numerical study reported in ref. 28).

Furthermore, SO coupling introduces the third “effective” lattice ~**Ω***σ*_3_ cos(2*γη*) and results in spatial modulation of the Rabi frequency ~**Ω***σ*_1_ sin(2*γη*). This explains a complex resonance-like structure of deeps in the *χ*(*γ, p*_2_) distribution at sufficiently large *γ* and *p*_2_ values. Moreover, the frequency of this additional effective potential in (7) is given by 2*γ*, and thus this potential can be averaged out in the limit *γ* → ∞. In this limit the equations for spinor components become decoupled again, the system behaves as two single-component ones with the chemical potentials *μ* ± Ω. For each of these systems the LDT threshold approaches the same value as the one encountered at *γ* = 0.

**Figure 3:**
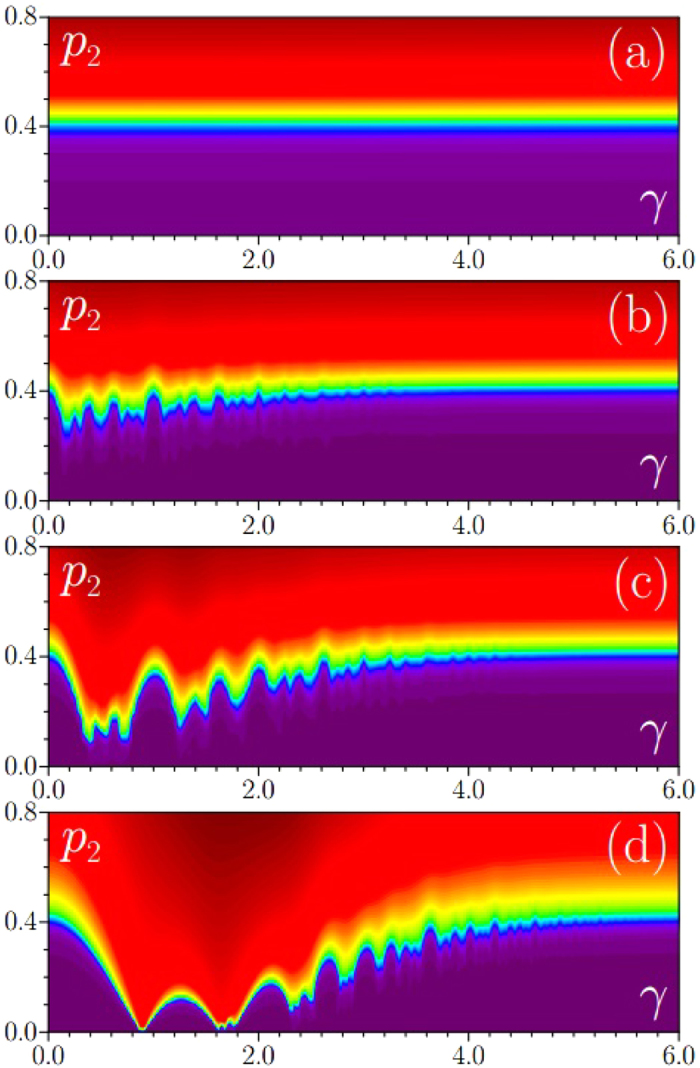
Form-factor of eigenmode with lowest chemical potential on the plane (*γ, p*_2_) at **Ω** = 0 (**a**), **Ω** = 0.01 (**b**), **Ω** = 0.1 (**c**), **Ω** = 1 (**d**). In all cases *p*_1_ = 1, *κ*_1_ = 2, *κ*_2_ = 5^1/2^ + 1.

Figure 3 shows transformation of the dependence *χ*(*γ, p*_2_) with increase of Rabi coupling **Ω**. As mentioned above, at **Ω** = 0 the system behaves as a single-component one and irrespectively of *γ* value the LDT occurs around *p*_2_ = 0.4. This “uniform” in *γ* dependence becomes gradually distorted with increase of the Rabi coupling. One can say that in general SO coupling acts toward reduction of LDT threshold, but this reduction is selective and occurs for particular values of *γ* around which band flattening occurs. Note that for **Ω** ≫ 1 the situation is possible where LDT threshold nearly vanishes within broad intervals of *γ*.

### Linear and nonlinear evolutions

**Figure 4:**
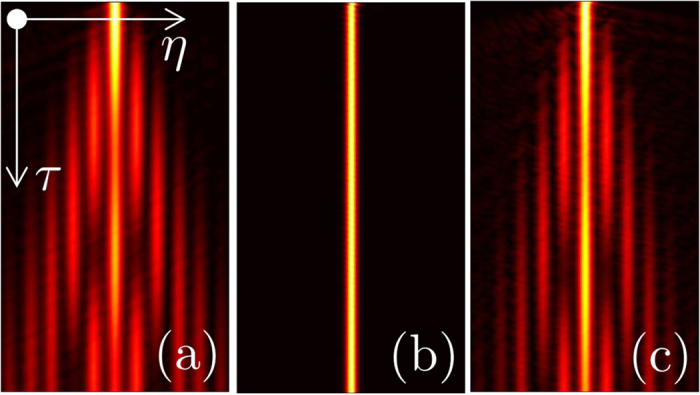
Evolution dynamics of narrow (initial width *w* = 1) Gaussian wavepackets with out-of-phase components at *γ* = 0.6 (**a**), *γ* = 0.9 (**b**), and *γ* = 1.2 (**c**) for *p*_1_ = 1, *p*_2_ = 0.1, and negligible inter- and intra-atomic interactions. Only density distribution |*ψ*_1_|^2^ of the first component is shown within the *η* ∈ [−17, +17] window and up to the time *τ* = 1200, since evolution of the second component is identical. In all cases Omega = 1.

The results based on the analysis of eigenmodes of the linear system are fully confirmed by solution of evolution Eq. (1) with initial Gaussian wavepackets (see Methods). Figure 4 shows representative evolution scenarios at p_1 = 1.0, p_2 = 0.1, and Omega = 1. As expected, when *γ* = 0.6 or *γ* = 1.2 the potential does not admit localized modes and any initial wavepacket disperses upon evolution (Fig. 4(a,c)). In contrast, at *γ* = 0.9 localized mode is excited and expansion of the central region of the wavepacket is quickly arrested (Fig. 4(b)).

**Figure 5:**
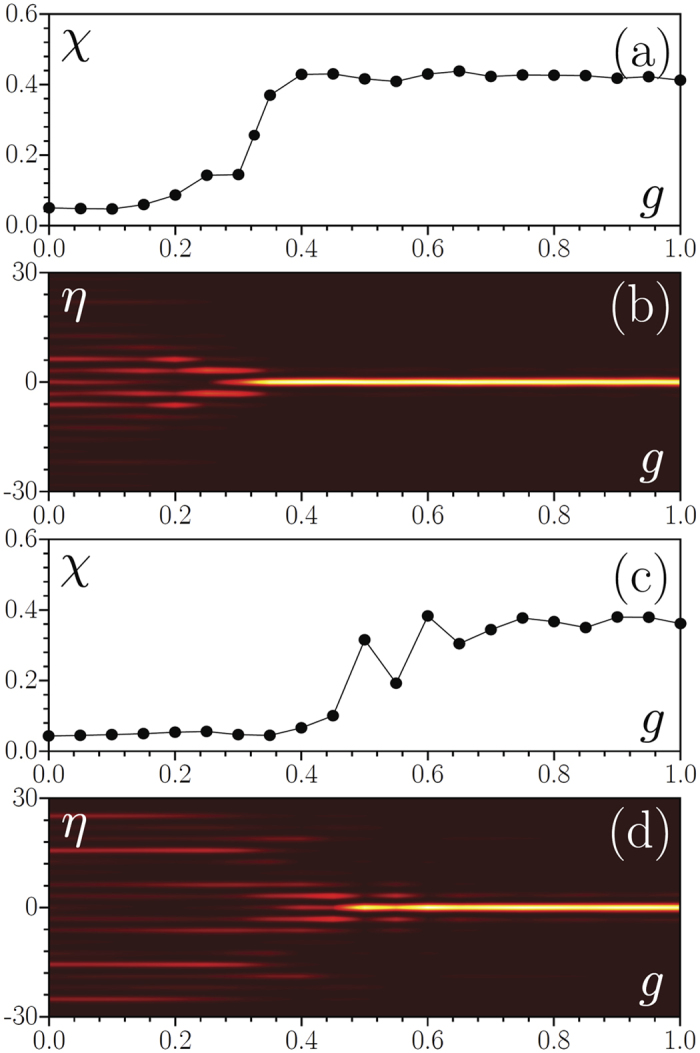
Crossover from delocalization to localization induced by growing repulsive interactions *g* for *p*_1_ = 1, *p*_2_ = 0.1 at *γ* = 0.6 and **Ω** = 1 (**a**,**b**), and at *γ* = 0 and **Ω** = 0 (**c**,**d**). (**a**,**c**) Form-factor of the wavepacket at *τ* = 2000 versus nonlinearity strength and (**b,d**) corresponding transformation of the output density distribution |*ψ*_1_|^2^. At *τ* = 0, both out-of-phase components have Gaussian shapes with *w* = 1 and unit amplitudes in both components.

Let us now turn to the effect of repulsive inter– and intra–atomic interactions, i.e. consider how increasing positive *g* > 0 (i.e. growing scattering length or total number of atoms) affects LDT. To this end, we use the same parameters and initial conditions as in Fig. 4 and solve Eqs. (1) taking into account repulsive interactions. We found that when *γ* = 0.9 (i.e. when localized modes exist already in linear system) the addition of repulsive interactions do not change qualitatively the output wavefunction: it remains localized even for *g* > 3 and increase of the scattering length results only in slight decrease of the maximal density. A completely different situation is observed when no localized modes exist in the linear system at *γ* = 0.6. In this case one observes relatively sharp crossover from dispersion at small values *g* to strong localization at large *g* (see Fig. 5(a,b), where we show final density distribution at *τ* = 2000 as a function of *g* and associated transformation of the form-factor) due to formation of matter-wave soliton, whose chemical potential resides somewhere within linear spectrum of delocalized modes (the existence of gap solitons in periodic SO coupled BECs with repulsive interactions was reported in refs 30, 31, 32). The critical value of *g* corresponding to delocalization-localization crossover depends on the SOC strength and the Rabi frequency, and in particular, in Fig. 5(c,d) we present such a crossover for the vanishing SO coupling and Rabi frequency. We note, however, that the LDT persists also in the absence of either SOC or Rabi coupling, or both. This becomes evident from the possibility of reducing Eq. (4) to nonlinearly coupled GPEs without linear coupling. Such equations admit one component solutions in which only one component is different from zero and hence are reduced to one-component Schrödinger equation with a bi-chromatic potential which features LDT. An example of this is given by the above transformation (6), (7), which either at **Ω** = 0 or at *γ* = 0 admits solutions **F** = (*f*_1_, 0)^*T*^ and **F** = (0, *f*_2_)^*T*^, with both *f*_1,2_ solving the nonlinear Schrödinger equation with a bi-chromatic lattice, and hence featuring LDT.

## Conclusions

In the present report we described several striking features of the localization-delocalization transition of a spin-orbit coupled BEC in an incommensurable bi-chromatic lattice. We have found that the moderate spin-orbit coupling drastically affects the phenomenon by reducing the intensity of one of the sub-lattices until very small values, what is explained by flattening of the lowest bands of the larger sub-lattice. It has been revealed that for observation of the phenomenon the Rabi coupling and the spin-orbit coupling must be present simultaneously. In the limit of the large spin-orbit coupling the system behaves like its one-component counterpart. We also found that increase of the inter-atomic positive scattering length results in localization of modes which are delocalized in the linear regime.

## Methods

Upon modeling of evolution of linear and nonlinear excitations in Eq. (1) we use standard split-step fast-Fourier approach that consists in splitting equation in several parts, each describing one particular effect, and their consecutive solution on sufficiently small time steps *dτ* = 0.001. For efficient excitation of the localized modes (if they exist) the selection of input conditions is crucial. Here we used two Gaussian wavepackets 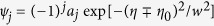 with *j* = 1, 2 and the width *w* = 1 for two spinor components. Their relative phase was taken to be *π* in accordance with the existence of *π* phase shift between components of lowest eigenmode (recall that in addition to this shift both components in stationary eigenmode feature nontrivial phase distributions). We also use small displacement *η*_0_ = 0.13 from the center of potential, since in this case emission of small-amplitude waves is strongly suppressed and localized modes are excited with high efficiency.

## Additional Information

**How to cite this article**: Li, C. *et al*. Localization-delocalization transition in spin-orbit-coupled Bose-Einstein condensate. *Sci. Rep.* **6**, 31700; doi: 10.1038/srep31700 (2016).
